# A multi-omics approach combining causal inference and *in vivo* validation identifies key protein drivers of alcohol-associated liver disease

**DOI:** 10.3389/fimmu.2025.1714502

**Published:** 2025-12-05

**Authors:** Qingyi Zhou, Xuan Ma, Qianqian Cui, Lei Zhang, Chao Yao, Zilu Zhang, Xiaoli Wang, Liang Chu

**Affiliations:** 1 Second Affiliated Hospital of Bengbu Medical University, Bengbu, China; 2 Bengbu Medical University, Bengbu, China; 3 Anhui Key Laboratory of Infection and Immunity of Bengbu Medical College, Bengbu, China; 4 School of Clinical Medicine, Shandong Second Medical University, Weifang, Shandong, China; 5 Department of Basic Medical College, Bengbu Medical University, Bengbu, China

**Keywords:** alcohol-associated liver disease, Mendelian randomization, SMR, pQTL, single-cell RNA sequencing, causal inference

## Abstract

**Background:**

Alcohol-associated liver disease (ALD) constitutes a global health crisis, yet the molecular mechanisms driving its pathogenesis remain unresolved, critically impeding the development of effective therapeutics. A fundamental challenge is the differentiation of correlational biomarkers from the causal drivers of disease. Here, we perform a systematic characterization of the ALD causal proteome to uncover novel pathogenic mediators and prioritize therapeutic targets.

**Methods:**

We implemented a multi-stage pipeline integrating human genetics with multi-level experimental validation. A two-sample Mendelian randomization (MR) framework, leveraging large-scale plasma proteomics and ALD GWAS data, was employed to nominate proteins causally linked to ALD. These candidates underwent functional enrichment analysis to delineate their biological roles. To rigorously control for confounding by genetic linkage, findings were validated using SMR. We then employed single-cell RNA sequencing from a murine ALD model to determine the hepatic cellular origins of the validated targets. Finally, their functional relevance was established *in vivo* using a chronic-plus-binge ethanol feeding mouse model.

**Results:**

Our MR analysis identified 17 proteins with a putative causal association with ALD. Functional enrichment analysis implicated these candidates in inflammatory and immune response pathways. After stringent SMR validation, we identified four high-confidence causal proteins: TREML2 (Triggering Receptor Expressed on Myeloid cells-like 2) and MMP12 (Matrix Metallopeptidase 12) as risk factors, and PLA2R1 (Phospholipase A2 Receptor 1) and MAX (MYC Associated Factor X) as protective factors. Single-cell transcriptomics resolved the cellular sources of these proteins within the liver, identifying hepatic myeloid cells (macrophages and monocytes) as the primary source of the risk-promoting TREML2 and MMP12. In contrast, the protective protein PLA2R1 was predominantly expressed in hepatocytes, while MAX exhibited broad expression. These genetic predictions were phenocopied in our ALD mouse model; hepatic expression of Treml2 and Mmp12 was significantly upregulated, whereas Pla2r1 and Max were downregulated, corroborating their respective roles in disease pathogenesis.

**Conclusion:**

By systematically integrating genetic causal inference with multi-level functional genomics, we identify and validate four causal protein drivers of ALD. Our findings unveil a novel pathogenic axis where ALD risk is governed by a balance between pro-inflammatory, matrix-remodeling myeloid cells (driven by TREML2/MMP12) and homeostatic hepatocyte functions (mediated by PLA2R1/MAX). These genetically validated targets provide critical insights into ALD pathophysiology and represent promising, mechanistically-defined avenues for therapeutic intervention.

## Introduction

Alcohol-associated liver disease (ALD) is a leading cause of morbidity and mortality from liver disease worldwide, posing a substantial public health burden (1–3). Globally, approximately 50% of cirrhosis-related mortality is attributable to excessive alcohol consumption (2). The clinicopathological spectrum of ALD is extensive, ranging from early-stage hepatic steatosis to more severe conditions such as alcoholic hepatitis, fibrosis, and cirrhosis, and in advanced stages increases the risk of hepatocellular carcinoma (4). Despite decades of research elucidating the downstream pathological events—such as metabolic reprogramming, oxidative stress, and sterile inflammation—therapeutic progress has been profoundly limited (5, 6). This stagnation stems from a fundamental knowledge gap: a failure to systematically distinguish the initial, causal triggers of disease from the myriad of downstream biomarkers that merely reflect ongoing tissue damage. This critical inability to pinpoint upstream causal drivers has critically impeded the development of targeted, effective therapies, leaving abstinence as the only disease-modifying intervention (7).

The post-genomic era has ushered in a paradigm shift for dissecting complex human diseases, moving beyond single-omics to an integrated, multi-omics approach. The recent technological maturation of high-throughput platforms (e.g., Olink, SomaScan) has enabled the large-scale measurement of circulating proteins, leading to the creation of vast protein quantitative trait loci (pQTL) maps (8, 9). By linking genetic variants to protein levels, these maps provide the essential tools for Mendelian randomization (MR)—a genetic epidemiological method that uses inherited genetic variants as instrumental variables to infer causal relationships between exposures (such as plasma protein levels) and disease outcomes, thus circumventing the confounding and reverse causation biases that plague traditional observational epidemiology (10, 11).

This proteogenomic strategy has already proven to be transformative across multiple medical disciplines. In cardiovascular medicine, it has identified novel causal proteins for coronary artery disease and heart failure, revealing new biological pathways and de-risking potential drug targets before clinical trials (12). Its utility has been rapidly confirmed in neurobiology, where it has untangled the complex etiology of Alzheimer’s disease by identifying specific plasma proteins that causally influence disease risk (13, 14). Furthermore, this approach is beginning to illuminate the causal architecture of cancer and autoimmune diseases, providing unprecedented insights into tumor biology and immune dysregulation (15–19). While significant progress has been made applying proteogenomic approaches to cardiovascular disease, neurodegeneration, and cancer, the causal proteome of ALD—a globally prevalent condition with limited therapeutic options beyond abstinence—remains uncharacterized. This represents a critical gap given the substantial public health burden and the urgent need for targeted therapeutic strategies.

We apply this state-of-the-art, multi-layered framework to systematically resolve the causal protein drivers of ALD. Our strategy employs a rigorous validation funnel, beginning with a comprehensive MR-based screening to identify high-confidence causal candidates from human population data. We then integrate these findings with transcriptomics and single-cell RNA sequencing data to refine causal signals and pinpoint their precise cellular origins within the liver microenvironment. We also provide crucial *in vivo* validation in a clinically relevant animal model.

The objective of this study is to systematically identify and validate plasma proteins causally linked to ALD pathogenesis through an integrated framework combining MR, SMR, single-cell transcriptomic profiling, and experimental validation in a murine ALD model. This multi-layered approach aims to distinguish true causal drivers from correlational biomarkers, providing mechanistically-defined therapeutic targets for ALD intervention.

## Materials and methods

### Study design

**Figure 1:**
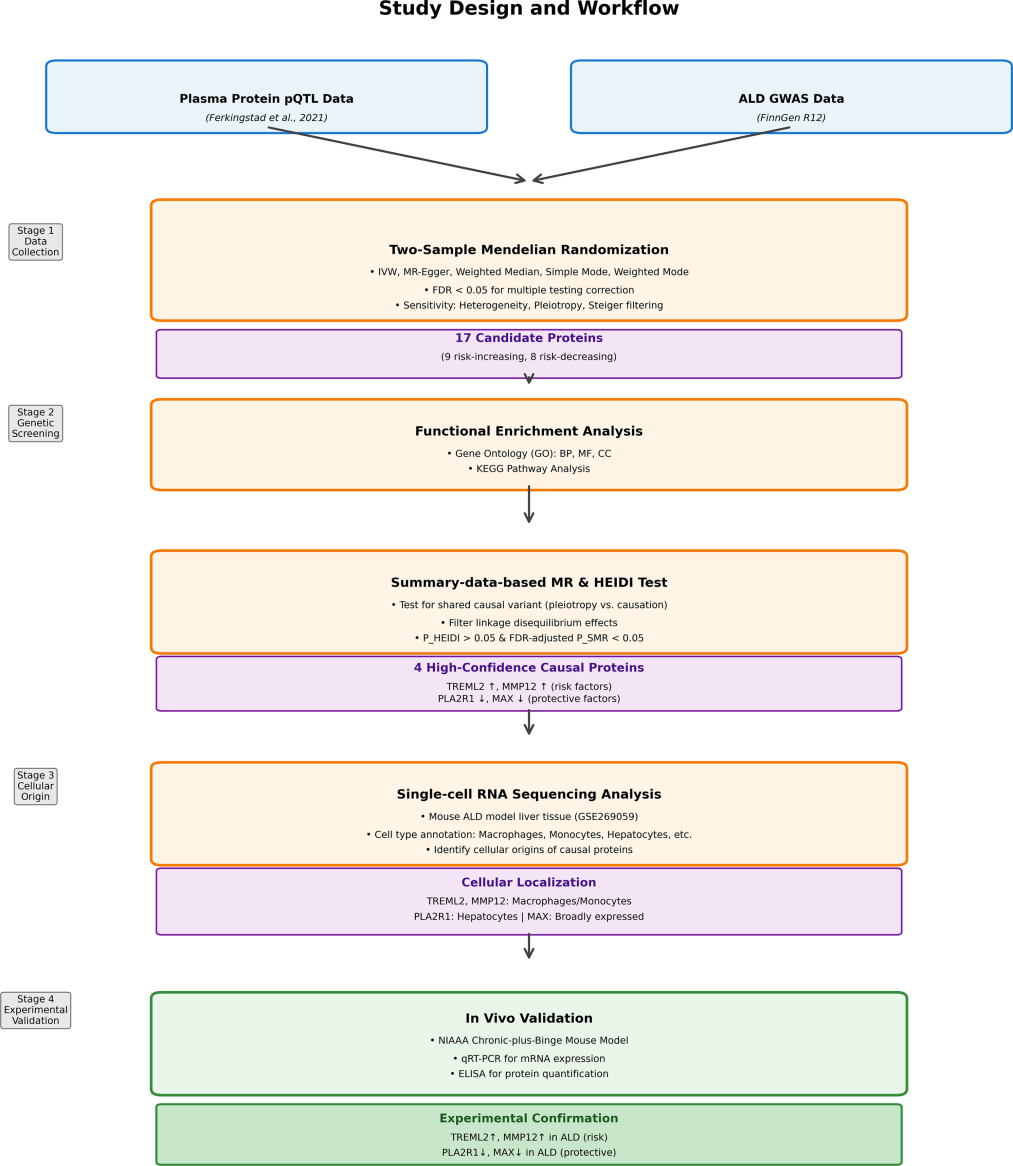
Study design.

This study used a multi-stage design to identify plasma proteins causally linked to ALD (**Figure 1**). This study was designed according to STROBE-MR guidelines to ensure the integrity of this MR study (**Supplementary Table S1**). We first performed a two-sample MR using large-scale pQTL and ALD GWAS data to screen for potential causal proteins. These candidates then underwent Gene Ontology (GO) and KEGG pathway analyses for functional annotation. To refine causal signals and mitigate linkage disequilibrium, we applied Summary-data-based MR with the HEIDI test. The cellular origins of these core proteins were identified using liver scRNA-seq data, followed by *in vivo* validation in a chronic-plus-binge mouse model. Detailed characteristics of the pQTL and GWAS datasets, including sample sizes, ancestry composition, measurement platforms, and data access information, are provided in **Supplementary Table S3**.

### GWAS data

We sourced summary statistics for plasma protein levels from a public database (20). Genetic variants were selected as potential instruments if they met a genome-wide significance threshold (p < 5 × 10^−8^). Instrument strength was rigorously evaluated for all genetic variants. For each protein, we calculated the F-statistic using the formula F = R²(N-K-1)/[K(1-R²)], where R² represents variance explained, N is sample size, and K is the number of instruments. We retained only instruments with F>10 to avoid weak instrument bias (**Supplementary Table S2**). These instruments were then pruned for linkage disequilibrium (R² < 0.001 in a 10,000 kb window). Summary-level data for ALD were obtained from the FinnGen consortium (release r12; https://r12.finngen.fi/), which included 3,769 cases and 485,213 controls of European ancestry. The use of publicly available data obviated the need for further ethical consent.

### Mendelian randomization

To infer a causal relationship, we applied five complementary MR models: inverse-variance weighted (IVW), MR-Egger, Weighted Median, Simple Mode, and Weighted Mode. Causal estimates were primarily derived from the IVW model (10). A total of 1078 plasma proteins with available pQTL instruments were tested in the MR analysis. A False Discovery Rate (FDR) threshold of < 0.05 was used to account for multiple comparisons over a nominal significance level of p < 0.05. All analyses were performed in R (v4.4.1) with the “TwoSampleMR” package. The validity of our results was further interrogated through several sensitivity tests (**Table 1**). We checked for heterogeneity among instruments and examined for potential horizontal pleiotropy using both the MR-Egger intercept and the MR-PRESSO outlier test (p > 0.05 indicating no significant pleiotropy). To ensure correct causal directionality, we performed Steiger filtering for all instruments using the ‘mr_steiger’ function in TwoSampleMR.

**Table 1:** Sensitivity analyses.

	Heterogeneity			Pleiotropy	
Exposure	Q	Q_df	Q_pval	Egger_intercept_pval	Global_Test_Pvalue
SEMA4D	94.19	72	0.04	0.255713662	0.055
MAX	8.48	12	0.75	0.913931331	0.802
TSTD1	14.57	16	0.56	0.249481672	0.578
PIP	31.44	47	0.96	0.124927716	0.958
NRP1	41.83	36	0.23	0.575262994	0.256
TREML2	95.11	90	0.34	0.449159764	0.332
ADH1C	5.32	7	0.62	0.503328828	0.604
LDLRAP1	1.09	3	0.78	0.437136986	0.762
TIE1	17.54	24	0.82	0.328987084	0.837
PLA2R1	140.43	115	0.05	0.098302172	0.06
LIFR	13.64	16	0.63	0.572755588	0.581
ARL3	3.64	10	0.96	0.901040202	0.975
MMP12	74.25	69	0.31	0.736527447	0.329
CST1	82.33	91	0.73	0.143125255	0.735
DPT	71.57	73	0.53	0.899261389	0.523
LUM	15.13	18	0.65	0.516861187	0.69
ST6GALNAC6	4.59	5	0.47	0.870146147	0.506

**Table 2:** Summary-data-based Mendelian randomization analysis.

probeID	ProbeChr	Gene	Probe_bp	topSNP	b_SMR	se_SMR	p_SMR	p_HEIDI	topSNP_F	p_SMR_FDR
ENSG00000125952	14	MAX	65472892	rs4902361	-0.42121	0.135232	0.00184114	0.4593112	466.3383433	0.02945824
ENSG00000262406	11	MMP12	102869953	rs72983568	0.173999	0.0626317	0.005467262	0.7218279	2494.033739	0.043738096
ENSG00000153246	2	PLA2R1	159997062	rs3749117	-0.0790182	0.0302958	0.009101324	0.9140036	25845.49745	0.045518784
ENSG00000112195	6	TREML2	41158015	rs13207171	0.130901	0.0533954	0.01422462	0.865941	7809.961002	0.045518784

### Enrichment analysis

Functional annotation of the prospective druggable gene set was performed through Gene Ontology (GO) and Kyoto Encyclopedia of Genes and Genomes (KEGG) pathway enrichment analyses, implemented with the ‘clusterProfiler’ R package (v4.10.1). This approach systematically characterized the involvement of these genes in key Biological Processes (BP), Molecular Functions (MF), and Cellular Components (CC), and identified their participation in significant metabolic and signaling pathways.

### SMR and HEIDI tests

A Summary-data-based Mendelian Randomization analysis (SMR), which tests whether the genetic associations between protein levels and ALD are driven by the same causal variant (supporting pleiotropy consistent with causation) rather than by linkage disequilibrium with distinct causal variants, was conducted using SMR software v1.3.1. The underlying SMR assumptions were evaluated using the Heterogeneity in Dependent Instruments (HEIDI) test to filter out associations resulting from horizontal pleiotropy. Genetic instruments were pruned based on the HEIDI test results (P-HEIDI < 0.05). A Benjamini–Hochberg correction was applied to the SMR P-values, with an adjusted P < 0.05 considered statistically significant.

### Single-cell analysis

Four genes passed these criteria: PLA2R1, TREML2 (Triggering Receptor Expressed on Myeloid cells-like 2), MMP12 (Matrix Metallopeptidase 12), and MAX (MYC Associated Factor X). We analyzed public single-cell RNA sequencing data (GSE269059) from a murine model of alcohol-associated liver disease (21). This dataset was selected because (1): it employed the same chronic-plus-binge ethanol feeding protocol (NIAAA model) that we used for *in vivo* validation, ensuring methodological consistency (2); it provides comprehensive hepatic cellular coverage with over 45,000 high-quality single cells spanning all major liver cell types (3); it represents one of the few publicly available scRNA-seq datasets specifically generated from an ALD model (rather than other liver injury models); and (4) the data quality metrics (median genes/cell 1,500, low mitochondrial content) met stringent standards for downstream analysis.

After filtering low-quality cells, gene expression data was log-normalized, and the top 1,500 highly variable genes were identified. The Harmony algorithm was used for data integration to correct for batch effects. Principal Component Analysis (PCA) was then performed, and the top 20 PCs were selected for downstream analysis, including unsupervised clustering with the Louvain algorithm. The t-SNE algorithm was used to visualize the Harmony-corrected data. Cell clusters were annotated into eight major cell types using the SingleR package and the expression of canonical marker genes. To investigate the expression patterns of specific genes, we visualized their expression levels using FeaturePlot and DotPlot functions.

### Animal models of ALD

The specific pathogen-free C57BL/6 J mice (8–10 weeks old with weight of 22–25 g)were purchased from the Experimental Animal Center of Bengbu Medical University. Alcohol-induced liver injury was produced using a chronic-plus-binge (NIAAA) paradigm: control Lieber–DeCarli liquid diet for adaptation, Lieber–DeCarli diet containing 5% (v/v) ethanol from days 1–10 (pair-fed controls received isocaloric maltodextrin), then a single ethanol gavage on day 11 (5 g/kg, 31.5% v/v in saline) between 09:00–10:00; controls received isocaloric maltodextrin. Necropsy occurred 9 h post-binge under isoflurane anesthesia (22). Blood was collected by cardiac puncture and serum isolated by centrifugation (1,500 g, 10 min, 4°C). Livers were dissected and allocated for downstream assays: formalin fixation (10% neutral-buffered formalin) for H&E, OCT-embedding and snap-freezing for Oil Red O, and snap-freezing in liquid N_2_ for RNA extraction. Samples were coded to preserve blinding. All animal experimental procedures comply with the ethical guidelines of Bengbu University and were approved by the Ethics Committee (Approval No. [2025]476).

### Serum biochemistry

Serum ALT and AST were measured using kinetic colorimetric assays on a clinical chemistry platform. The analyses were performed according to the standard operating procedures of the instrument, and internal quality controls were run concurrently.

### Hematoxylin–eosin staining

For histological analysis, frozen liver sections were fixed and stained with hematoxylin and eosin (H&E) using standard protocols. Stained sections were subsequently dehydrated in graded ethanol, cleared with xylene, and mounted with a neutral mounting medium. Images were acquired using a Nikon ECLIPSE E100 microscope.

### Oil Red O staining

To evaluate hepatic lipid accumulation, frozen liver sections were fixed and stained with Oil Red O solution, followed by hematoxylin counterstaining to visualize nuclei. Sections were then mounted with glycerol gelatin and imaged using a light microscope.

### RNA extraction and quantitative PCR

Total RNA was extracted from approximately 50 mg of liver tissue using TransZol Up (TransGen Biotech, Beijing, China). Following quantification, total RNA was reverse-transcribed into cDNA using TransScript^®^ SuperMix which includes a genomic DNA remover (TransGen Biotech), in accordance with MIQE guidelines (23). qPCR was performed in technical triplicate using PerfectStart^®^ Green qPCR SuperMix (TransGen Biotech). The stability of the reference gene (Gapdh) was validated across all experimental groups. Primer sequences are listed in **Table 3**. Relative gene expression was calculated using the 2^−ΔΔCt^ method, with efficiency correction applied where applicable.

**Table 3:** Primer sequences.

Primer	Forword primer sequence	Reverse primer sequence
PLA2R1	CCTGATGAAAAGATCGTGGAGAG	AAATGAAGAGACAACAGGTGACC
MAX	ACCATAATGCACTGGAACGAAA	GTCCCGCAAACTGTGAAAGC
MMP12	CTGCTCCCATGAATGACAGTG	AGTTGCTTCTAGCCCAAAGAAC
TREML2	AGAACCGCCGGAACTTAGTTG	TGATACGGACTACCTTGACCTTG

### Enzyme-linked immunosorbent assay

Hepatic protein levels of MAX, MMP12, PLA2R1, and TREML2 were quantified using commercial ELISA kits according to the manufacturer’s protocols. Liver tissue lysates were diluted to appropriate concentrations and assayed in duplicate using mouse-specific ELISA kits(BYabscience, Nanjing, China) for MAX, MMP12, PLA2R1, and TREML2. Absorbance was measured at 450 nm using a microplate reader, and protein concentrations were determined from standard curves. Results were normalized to total protein content and expressed as ng/mg total protein.

### Statistical analysis

All statistical analyses were conducted using GraphPad Prism 8.0. After assessing for normality with the Shapiro-Wilk test, differences between the two groups were determined by an unpaired, two-tailed Student’s t-test (for normally distributed data) or a Mann-Whitney U test (for non-normally distributed data). A P-value < 0.05 was considered significant.

## Results

### Mendelian randomization analysis

**Figure 2:**
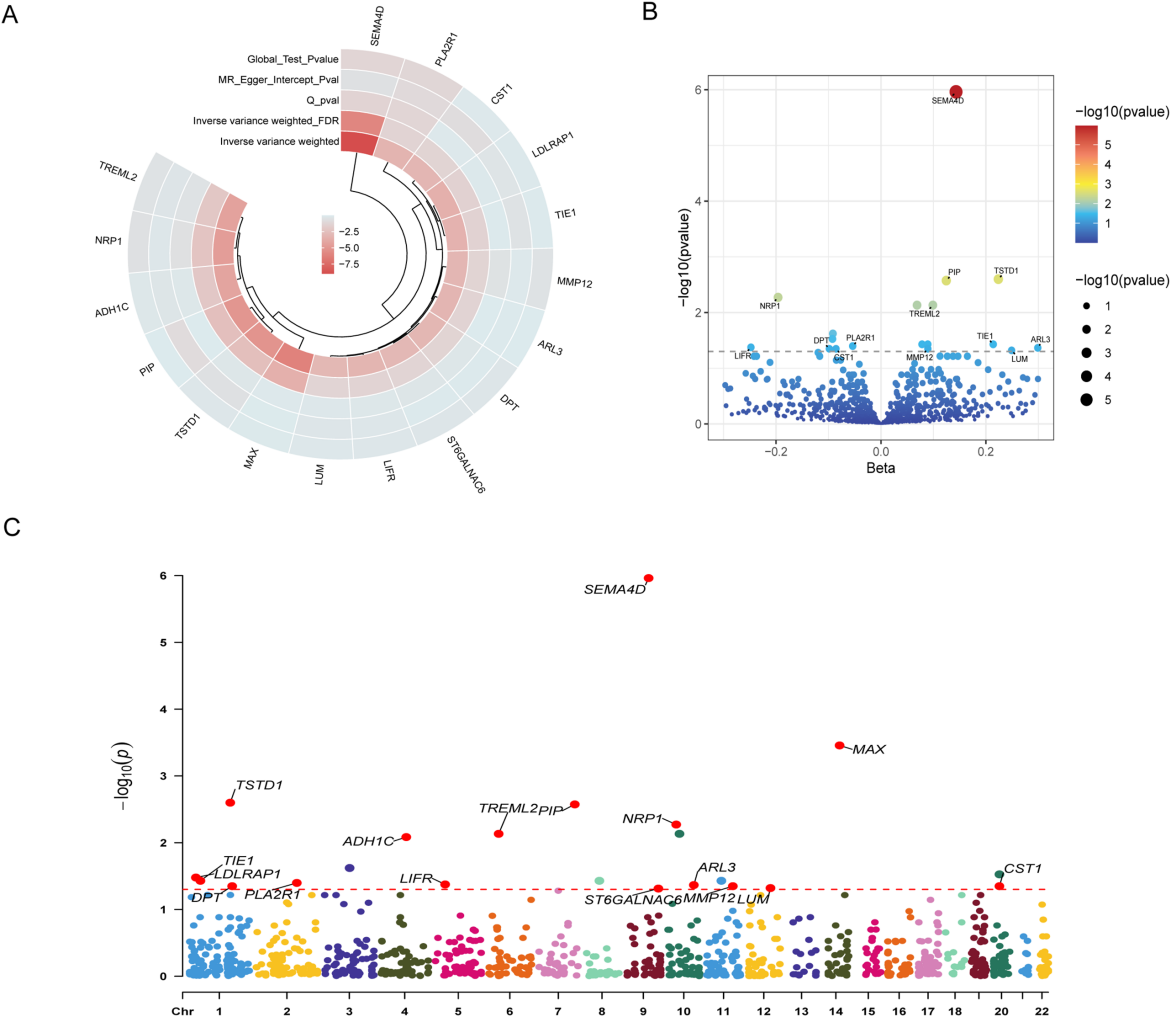
Mendelian randomization analysis identifies 17 plasma proteins causally associated with ALD. **(A)** A Circos plot summarizing the MR analysis results for the 17 proteins that passed the FDR threshold. The rings, from inside to outside, represent (1): the -log10(p-value) of the primary inverse-variance weighted analysis, with darker red indicating higher significance (2); the inverse-variance weighted FDR (3); the p-value for Cochran’s Q test for heterogeneity (4); the p-value for the MR-Egger intercept test for directional pleiotropy; and (5) the p-value for the MR-PRESSO global test for outlier-driven pleiotropy. **(B)** A volcano plot illustrating the causal effect size versus statistical significance for all tested proteins. **(C)** A Manhattan plot displaying the genomic location of the lead instrumental variables (SNPs) for each protein across all 22 autosomes.

Using IVW as the primary estimator, we evaluated the causal relevance of genetically proxied plasma proteins for ALD. The results of this analysis are summarized in **Figure 2**. Of the proteins tested, 17 remained significant after multiple-testing control (FDR < 0.05), as displayed in the summary Circos plot and volcano plot (**Figure 2A, B**). These proteins were: SEMA4D, MAX, TSTD1, PIP, NRP1, TREML2, ADH1C, LDLRAP1, TIE1, PLA2R1, LIFR, ARL3, MMP12, CST1, DPT, LUM, and ST6GALNAC6. Instrument strength for the proteins was consistently robust, with mean F-statistics ranging from 51.8 (ST6GALNAC6) to 600.2 (PLA2R1), all substantially exceeding the threshold of 10. The proportion of variance explained (Mean_R²) ranged from 0.14% to 1.6%, indicating instruments of adequate strength across all prioritized proteins (**Supplementary Table S2**).

As depicted in the result, these associations could be divided into risk-increasing and risk-decreasing effects. Nine proteins showed risk-increasing associations: SEMA4D (OR 1.154, 95% CI 1.102–1.208; FDR = 1.1×10^-6^), TSTD1 (1.251, 1.134–1.379; 0.003), PIP (1.133, 1.072–1.197; 0.003), TREML2 (1.104, 1.053–1.158; 0.007), ARL3 (1.348, 1.135–1.602; 0.043), MMP12 (1.094, 1.038–1.152; 0.045), TIE1 (1.239, 1.099–1.396; 0.037), LUM (1.283, 1.107–1.487; 0.048), and ST6GALNAC6 (1.816, 1.273–2.591; 0.049). Conversely, eight proteins were associated with a decreased risk of ALD: MAX (0.637, 0.533–0.761; 3.5×10^-4^), NRP1 (0.822, 0.750–0.900; 0.005), ADH1C (0.641, 0.516–0.797; 0.008), LDLRAP1 (0.544, 0.390–0.759; 0.033), PLA2R1 (0.948, 0.919–0.977; 0.040), LIFR (0.780, 0.677–0.900; 0.042), CST1 (0.918, 0.873–0.965; 0.045), and DPT (0.906, 0.855–0.960; 0.045). The Manhattan plot illustrates that these associations map to distinct chromosomal coordinates, supporting that the observed effects arise from independent loci rather than linkage among correlated variants(**Figure 2C**). Steiger directionality analysis confirmed the assumed causal direction for the FDR-significant proteins (Steiger p < 0.001 for all proteins), providing confidence that observed associations reflect effects of protein levels on disease risk rather than reverse causation or confounding (**Supplementary Table S2**).

### Functional enrichment analysis

**Figure 3:**
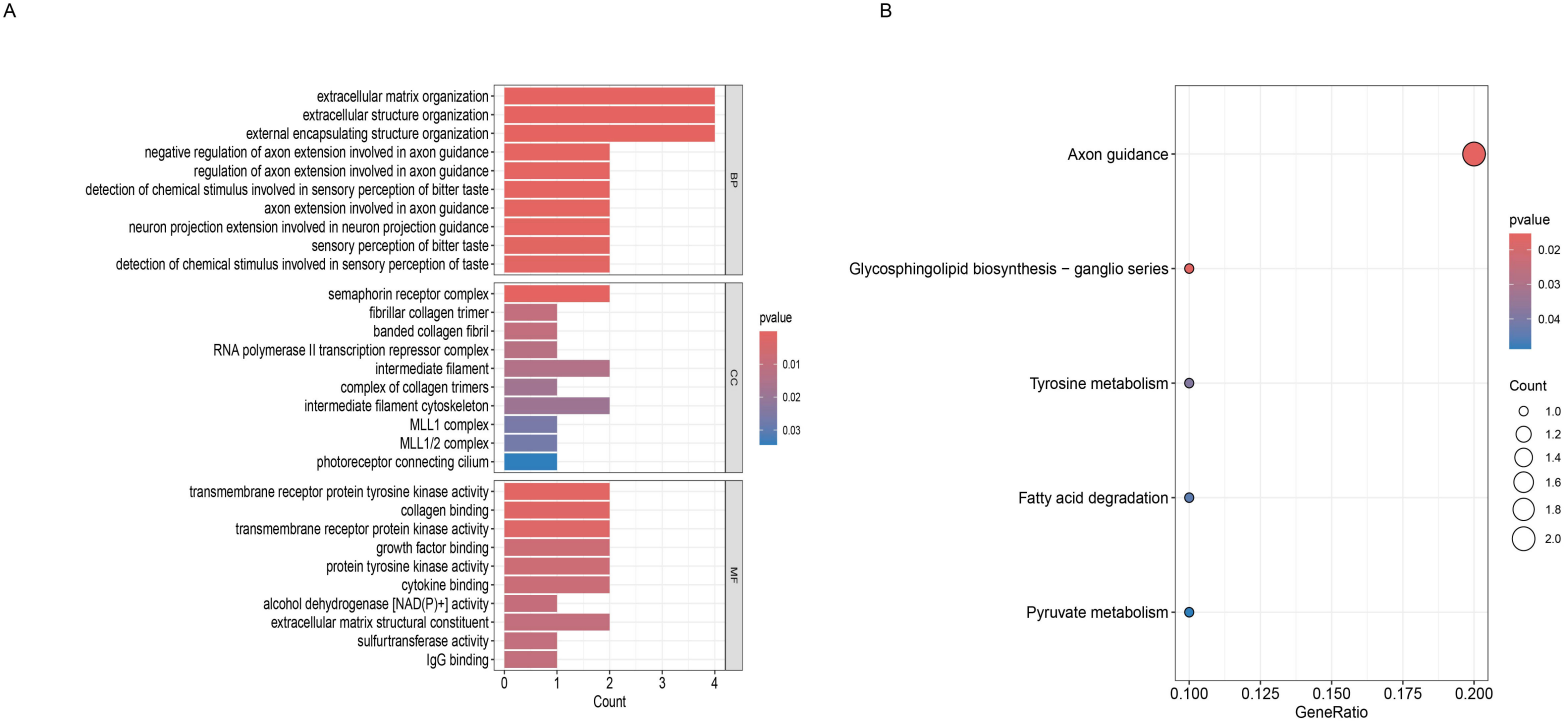
Functional enrichment analysis reveals pathways associated with ALD-risk proteins. **(A)** GO enrichment analysis of the 17 ALD-associated proteins. **(B)** KEGG pathway enrichment analysis. The bubble plot shows the most significant pathways.

To elucidate the biological roles of the 17 candidate proteins causally associated with ALD, we performed functional enrichment analysis. Gene Ontology (GO) analysis revealed that these proteins were predominantly involved in processes related to extracellular matrix (ECM) dynamics and neural development (**Figure 3A**). Specifically, the most significantly enriched Biological Process (BP) terms included “extracellular matrix organization” and “extracellular structure organization.” Multiple terms related to axon development, such as “regulation of axon extension involved in axon guidance,” were also highly significant. Consistent with these processes, key Molecular Function (MF) terms included “extracellular matrix structural constituent,” “collagen binding,” and “transmembrane receptor protein tyrosine kinase activity.” The Cellular Component (CC) analysis localized these proteins to relevant compartments, including the “fibrillar collagen trimer” and the “semaphorin receptor complex.”

To further explore the systems-level functions of these proteins, we conducted a KEGG pathway analysis (**Figure 3B**). This analysis strongly corroborated the GO findings, identifying “Axon guidance” as the most significantly enriched pathway, both in terms of gene ratio and statistical significance. Additionally, the analysis highlighted a cluster of dysregulated metabolic pathways, including “Fatty acid degradation,” “Pyruvate metabolism,” and “Tyrosine metabolism,” underscoring a potential link between the identified proteins and metabolic reprogramming in ALD. These results identify a coherent functional signature for the ALD-associated proteins, implicating them in the intersection of tissue remodeling, neural-related signaling, and metabolic dysfunction.

### Summary-data–based Mendelian randomization analysis

We applied SMR analysis with HEIDI testing to the MR-prioritized proteins, retaining associations that met stringent criteria (p_HEIDI>0.05 and FDR-adjusted p_SMR < 0.05). Four proteins passed validation (**Table 2**): PLA2R1, TREML2, MMP12, and MAX. All instruments demonstrated strong genetic signals (F-statistics 466–25,845), utilized 8–20 independent SNPs for heterogeneity testing, and showed no evidence of pleiotropy (p_HEIDI = 0.459–0.914), supporting a shared causal variant underlying both the pQTL and ALD risk signals. Among the validated proteins, PLA2R1 and MAX exhibited protective effects (OR = 0.92 and 0.66, respectively), while TREML2 and MMP12 conferred increased risk (OR = 1.14 and 1.19, respectively), with MAX demonstrating the strongest effect magnitude. The effect sizes and consistency across MR methods for the four SMR-validated proteins are visualized in forest plots (**Supplementary Figure S1**).

### Single-cell analysis

**Figure 4:**
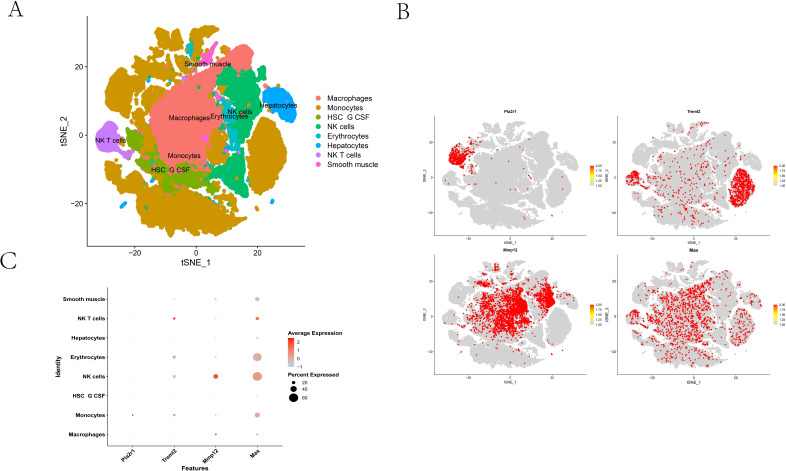
Single-cell analysis identifies the cellular sources of ALD-associated proteins in the mouse liver. **(A)** A t-SNE plot showing the major cell populations identified from single-cell RNA sequencing of liver tissue from ALD mice. **(B)** Feature plots illustrating the normalized expression of the four genes (Pla2r1, Treml2, Mmp12, and Max) projected onto the t-SNE manifold. The color scale represents the level of gene expression. **(C)** A dot plot summarizing the expression of the four feature genes across the identified cell types.

To identify the cellular sources of the ALD-associated proteins, we analyzed a recently published single-cell RNA sequencing dataset (GSE269059) from the livers of mice subjected to chronic-plus-binge ethanol feeding (21). Unsupervised clustering and annotation resolved eight major cell compartments: Macrophages, Monocytes, HSC/G-CSF cluster, NK cells, NK T cells, Hepatocytes, Smooth muscle, and Erythrocytes, all of which formed distinct clusters in t-SNE space (**Figure 4A**). We next projected the four SMR-prioritized genes (Pla2r1, Treml2, Mmp12, Max) onto this cellular map. The resulting feature maps revealed that Mmp12 and Treml2 expression was concentrated almost exclusively within the innate immune populations, co-localizing with the Macrophage and Monocyte clusters. In contrast, Pla2r1 expression was predominantly localized to the parenchymal (Hepatocyte) territories, with only sparse signal in immune cells. Max, a ubiquitous transcriptional regulator, displayed broadly distributed expression with modest heterogeneity across all compartments (**Figure 4B**). A dot plot summary corroborated these spatial patterns at the cell-type level (**Figure 4C**). Mmp12 and Treml2 exhibited the largest dots (higher “percent expressed”) and the highest average expression in Macrophages and Monocytes, with minimal signal in hepatocytes and other non-myeloid lineages. Conversely, Pla2r1 showed its highest average expression in Hepatocytes. Max was widely expressed across most cell types, consistent with its role as a core transcriptional regulator. Together, these data nominate myeloid cells (Macrophages and Monocytes) as the principal hepatic source for the two risk-increasing proteins (TREML2 and MMP12), while hepatocytes primarily account for the risk-decreasing PLA2R1 signal, and the MAX signal is broadly distributed. These cell-of-origin assignments are internally consistent across both the manifold overlays and the dot-plot aggregates and provide a critical cellular context for the causal relationships inferred by MR analysis.

Notably, while Max exhibited broad expression across most cell types, a distinct subset of NK-T cells (~15–20%) demonstrated particularly high *Max* expression (**Figure 4C**). This observation is intriguing given the complex and context-dependent roles of NK-T cells in liver disease, which can exert both cytotoxic (pro-injury) and immunoregulatory (anti-inflammatory) functions depending on their activation state and cytokine milieu.

### Chronic alcohol administration induces significant liver injury and steatosis in mice

**Figure 5:**
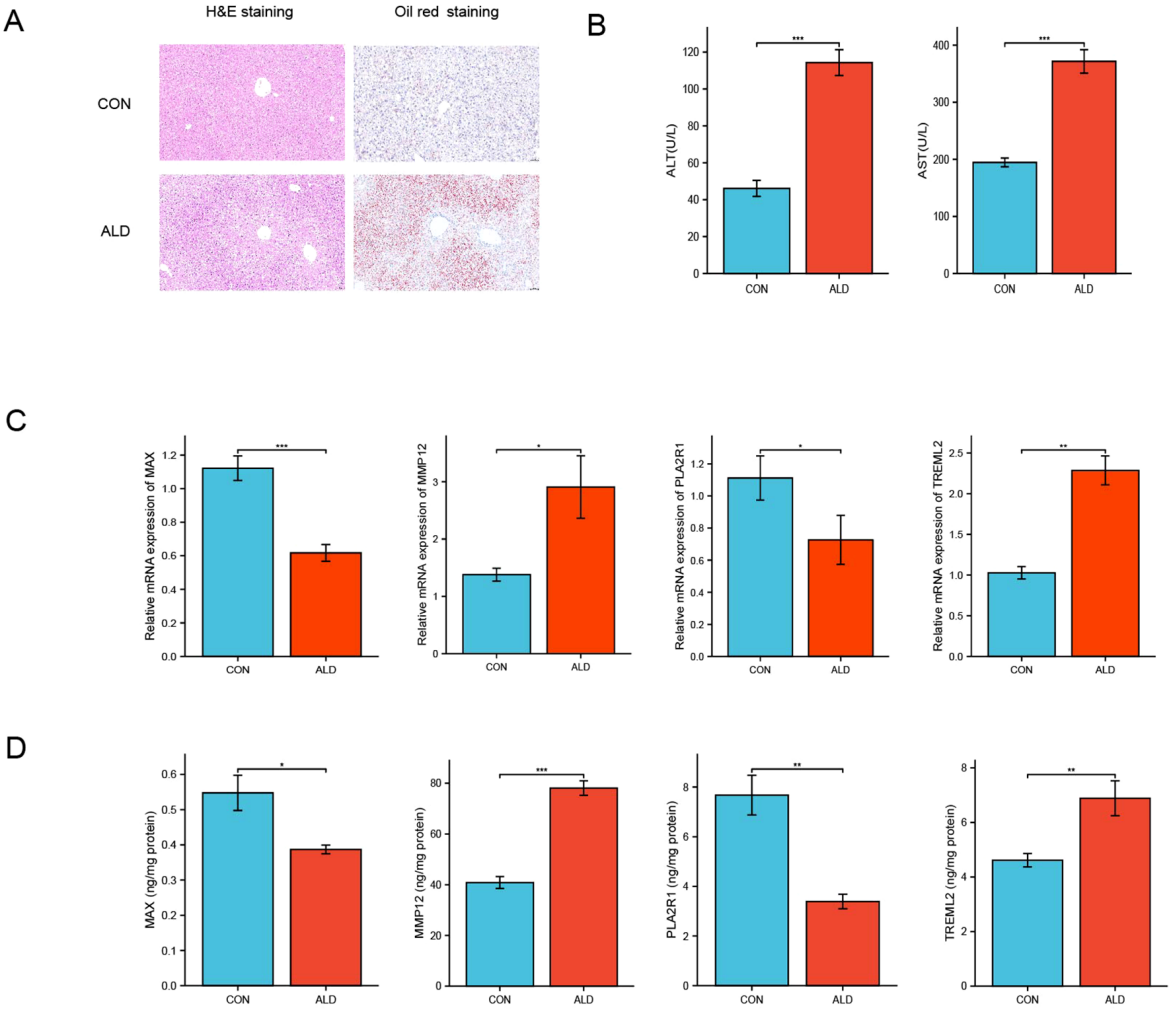
Validation of alcohol-induced liver injury and associated gene expression changes in a murine model. **(A)** Representative histological images of liver sections from control (CON) and ALD mice stained with H&E and Oil Red O (scale bars = 100 μm). **(B)** Quantification of serum liver injury markers, ALT and AST (U/L). **(C)** Relative mRNA expression levels of MAX, MMP12, PLA2R1, and TREML2 in liver tissue from CON and ALD mice, measured by qRT-PCR and normalized to GAPDH. **(D)** Hepatic protein levels of MAX, MMP12, PLA2R1, and TREML2 quantified by ELISA and normalized to total protein content (ng/mg protein). Statistical significance was determined using unpaired two-tailed Student’s t-test. p < 0.05, p < 0.01, p < 0.001. *P < 0.05, **P < 0.01, ***P < 0.001.

To validate the causal relationships suggested by our MR analysis, we first established a murine model of ALD. Histopathological changes in the liver were examined by histological staining (**Figure 5A**). Hematoxylin and eosin (H&E) staining revealed marked abnormalities in the liver architecture of the ALD group compared to the control group. These changes were characterized by disordered hepatocyte arrangement and prominent hepatocyte swelling (ballooning degeneration). Furthermore, Oil Red O staining demonstrated a substantial and diffuse accumulation of red lipid droplets in the ALD group, indicating the development of severe hepatic steatosis, which was nearly absent in the control group. To quantify the extent of liver damage, key serum markers were measured (**Figure 5B**). Biochemical analysis confirmed that the serum levels of both ALT and AST were significantly elevated in the ALD group compared to the control group (p < 0.001). These findings collectively confirm the successful induction of severe hepatocellular injury by chronic alcohol administration.

### Transcriptional dysregulation of candidate genes in alcoholic liver disease

To validate the causal relationships identified by MR analysis, we quantified the hepatic expression of MAX, MMP12, PLA2R1, and TREML2 using qRT-PCR (**Figure 5C**). At the mRNA level, MAX and PLA2R1 were significantly downregulated in ALD livers compared to controls (p < 0.001 and p < 0.05, respectively), while MMP12 and TREML2 showed significant upregulation (p < 0.05 and p < 0.01, respectively).

### Protein-level validation confirms directional consistency

Protein quantification by ELISA fully recapitulated these transcriptional changes (**Figure 5D**). MAX and PLA2R1 protein levels were significantly reduced in ALD livers (p < 0.05 and p < 0.01, respectively), whereas MMP12 and TREML2 were markedly elevated (p < 0.001 and p < 0.01, respectively). These findings demonstrate concordant dysregulation of all four proteins at both mRNA and protein levels, with directionality precisely matching our genetic predictions, thereby providing robust experimental validation of their causal involvement in ALD pathogenesis.

## Discussion

In this study, we employed a multi-stage pipeline integrating Mendelian randomization, SMR, single-cell transcriptomics, and *in vivo* validation to dissect the complex causal proteome of ALD. This robust, genetics-led approach successfully navigated the challenges of confounding and reverse causality inherent in observational studies, prioritizing 17 plasma proteins with putative causal links to ALD. Subsequent stringent filtering with SMR analysis distilled this list to four high-confidence candidates: PLA2R1, TREML2, MMP12, and MAX. Our findings not only identify these proteins as novel players in ALD pathogenesis but also assign them to distinct cellular compartments within the liver, offering a cohesive mechanistic framework that links myeloid cell-driven inflammation with hepatocyte-specific responses.

A key finding of our study is the opposing roles and distinct cellular origins of the identified risk and protective proteins. The two risk-increasing proteins, TREML2 and MMP12, were predominantly expressed in hepatic myeloid cells (macrophages and monocytes) (24). This strongly implicates the innate immune system as a primary driver of ALD pathology, a concept well-supported by existing literature demonstrating that macrophage activation is a central event in alcoholic hepatitis (24). TREML2 (Triggering Receptor Expressed on Myeloid cells-like 2) belongs to the TREM family of immunoreceptors (25). Its close relative, TREM-1, is known to amplify TLR-mediated inflammatory signaling and is strongly implicated in both experimental ALD and human liver fibrosis (26).

While TREML2 and TREM2 share structural homology as members of the TREM immunoreceptor family, they exhibit critical functional distinctions. Unlike TREM2, which signals through the immunoreceptor tyrosine-based activation motif (ITAM) adapter DAP12 to promote an anti-inflammatory, restorative macrophage phenotype during liver injury resolution (27–29), TREML2 lacks a transmembrane lysine residue required for DAP12 binding and instead signals through alternative pathways (25). Emerging evidence suggests TREML2 may function as a pro-inflammatory amplifier by modulating NLRP3 inflammasome activation and enhancing microglial pro-inflammatory polarization in neuroinflammatory conditions (25). While the precise ligand for TREML2 remains unidentified—in contrast to TREM2, which binds phospholipids and apoptotic cell debris—structural modeling suggests TREML2 may recognize distinct damage-associated molecular patterns (DAMPs) released during hepatocyte injury. This differential ligand specificity and signaling architecture could explain why TREML2 promotes ALD pathogenesis through sustained inflammatory signaling, whereas TREM2 facilitates injury resolution. Our causal genetic evidence uniquely implicates TREML2 as a disease driver, suggesting that therapeutic strategies targeting TREML2 may offer benefits distinct from TREM2 modulation. Future studies should prioritize identification of TREML2 ligands in the ALD liver microenvironment and characterization of its downstream signaling cascades in hepatic macrophages. Our identification of TREML2 as a causal risk factor highlights the nuanced and critical balance of TREM signaling in dictating macrophage function and disease outcomes in the liver (30).

The second risk-associated protein, MMP12 (Matrix Metallopeptidase 12), also known as macrophage elastase, reinforces the central role of myeloid cells in tissue remodeling during liver injury (31). MMPs are critical mediators of extracellular matrix (ECM) turnover, and their dysregulation is a hallmark of liver fibrosis (32). Macrophage-derived MMP12 has been shown to regulate elastin degradation and contribute to fibrosis progression in multiple organs (31, 33). Our data provide direct causal evidence linking genetically elevated MMP12 levels to increased ALD risk, suggesting that excessive macrophage-led matrix degradation or remodeling actively contributes to the pathogenesis, potentially by releasing profibrotic signals or altering the structural integrity of the liver sinusoids.

Conversely, our study identified two proteins, PLA2R1 and MAX, whose genetically higher levels are protective against ALD. Our single-cell analysis localized the protective PLA2R1 signal primarily to hepatocytes. PLA2R1 (Phospholipase A2 Receptor 1) is a transmembrane receptor that acts as a critical regulator of cellular stress responses, with its function appearing highly context-dependent (34). While best known as the autoantigen in membranous nephropathy, its role in cellular homeostasis is complex and multifaceted. In the liver, recent evidence strongly supports a protective function. Supporting this protective role, experimental studies demonstrate that hepatocyte-specific deletion of PLA2R1 in mice exacerbates diet-induced metabolic dysfunction, inflammation, and steatosis, confirming its function as a guardian of hepatocyte metabolic health under lipotoxic stress (34, 35). This mechanistic evidence directly supports our genetic finding that higher PLA2R1 levels reduce ALD risk, likely by mitigating alcohol-induced hepatocyte lipotoxicity—a key pathogenic mechanism in early-stage ALD. Our finding that higher PLA2R1 levels are protective against ALD aligns perfectly with this, suggesting a primary mechanism where it mitigates alcohol-induced lipotoxicity and inflammation by clearing harmful secretory phospholipases A2 (sPLA2s). This protective role contrasts with findings in other biological contexts where PLA2R1 has been shown to promote cellular senescence. For instance, studies have demonstrated that PLA2R1 can mediate premature aging phenotypes and drive detrimental lung cell senescence in obstructive lung disease (36). This pro-senescent activity can also be beneficial, as PLA2R1-mediated senescence has been shown to inhibit spontaneous tumor formation during aging by eliminating damaged cells (36, 37). This functional dichotomy suggests that PLA2R1 acts as a cellular sensor, and the outcome of its activation—either protection or senescence—depends on the specific cell type and the nature of the stressor. We propose that in hepatocytes under alcohol-induced metabolic stress, the dominant function of PLA2R1 is to maintain homeostasis by sequestering pro-inflammatory sPLA2s. This protective clearance mechanism likely outweighs its potential pro-senescent signaling, leading to a net beneficial effect and reduced risk for ALD.

The strongest protective signal was observed for MAX (MYC Associated Factor X), a ubiquitously expressed core transcription factor. MAX forms heterodimers with the MYC family of proteins, which are master regulators of cell proliferation, metabolism, and apoptosis (38, 39). The MYC oncogene itself is frequently up-regulated in liver disease, where it drives metabolic reprogramming and contributes to fibrosis and hepatocellular carcinoma (40, 41). The protective effect of MAX is intriguing. MAX lacks a transcriptional activation domain and its heterodimerization with other proteins (like the MAD family) can antagonize MYC-driven transcription (42). Therefore, higher levels of MAX may exert a protective effect by competing with MYC, thereby dampening its pathological pro-proliferative and pro-metabolic signaling in response to alcohol-induced cellular stress.

Our *in vivo* validation in a murine ALD model corroborates the directionality of our human genetic findings. The observed upregulation of TREML2 and MMP12 alongside the downregulation of PLA2R1 and MAX in the livers of alcohol-fed mice provides a powerful biological link between the genetically predicted risk and the actual molecular response to alcohol exposure. This convergence of evidence from human genetics and animal models strongly supports the clinical relevance of these four proteins. This hepatocyte-myeloid cell crosstalk is a recognized driver of ALD, where damaged hepatocytes release signals that recruit and activate immune cells, which in turn exacerbate hepatocyte injury, potentially contributing to subsequent fibrogenesis if injury persists. Our results provide specific, causally-validated molecular players within this pathological loop.

Our single-cell analysis unexpectedly revealed elevated MAX expression in a subset of hepatic NK-T cells, a cell population with complex roles in ALD pathogenesis. NK-T cells can promote liver injury through cytotoxic granule release and IFN-γ production, but certain subsets also exhibit immunoregulatory functions that limit inflammation and fibrogenesis (43–46). Specifically, type I NKT cells have been shown to exacerbate alcoholic liver injury by producing pro-inflammatory cytokines (IL-4, IL-13) and activating hepatic stellate cells, whereas certain regulatory NKT subsets can suppress HSC activation and attenuate fibrosis through IFN-γ production and direct cytotoxicity against activated HSCs (44, 47, 48). MAX, as a transcriptional regulator, may influence NK-T cell effector function by modulating metabolic programs or cytokine production. Given that our genetic evidence indicates a protective role for MAX, we speculate that high MAX expression in this NK-T subset may promote a regulatory phenotype that dampens excessive immune activation during alcohol-induced injury. Alternatively, MAX may be upregulated as a compensatory response to limit MYC-driven proliferative/metabolic signaling in activated NK-T cells. Future studies employing NK-T cell-specific MAX perturbation or high-resolution single-cell functional assays (e.g., CITE-seq combining transcriptomics with surface protein profiling) are needed to define the functional consequences of MAX expression in this lymphocyte subset and its contribution to overall ALD pathogenesis. Importantly, the protective signal we observe at the population level suggests that the net effect of MAX—integrating its functions across hepatocytes, myeloid cells, and NK-T cells—is risk-lowering.

While our genetics-led approach provides high-confidence causal candidates, we acknowledge its limitations. Our study relies on pQTLs for circulating plasma proteins, and while we used scRNA-seq to infer tissue origin, the local protein concentrations within the liver microenvironment and post-translational modifications may be more directly relevant to pathophysiology. Furthermore, the Mendelian randomization framework operates on several core assumptions; although our use of SMR analysis helps mitigate horizontal pleiotropy by testing for a shared causal variant, residual or uncharacterized pleiotropic effects cannot be entirely excluded. An important limitation of our validation approach is the use of the NIAAA chronic-plus-binge ethanol feeding model, which effectively recapitulates early-stage ALD pathology—including hepatic steatosis, hepatocellular injury (elevated transaminases), and inflammatory infiltration—but does not reproduce the chronic fibrotic remodeling or cirrhotic architectural distortion observed in advanced human ALD. Finally, while our genetic and tissue-level validation data establish MAX as a causal factor in ALD protection, the precise molecular mechanisms—including potential antagonism of MYC signaling, modulation of other bHLH-Zip transcription factor networks, or cell-type-specific functions—remain to be elucidated through targeted functional studies. Future investigations employing conditional MAX knockout or overexpression models, combined with transcriptomic and chromatin profiling, will be essential to definitively establish these mechanistic details. These limitations, however, pave the way for clear future directions. The definitive test of our proposed model will require the development and analysis of cell type-specific conditional knockout mice subjected to chronic-plus-binge alcohol feeding models. Mechanistically, future studies should investigate the precise downstream signaling pathways modulated by these proteins. The prioritization of these targets via human genetics, however, represents a highly promising avenue for future ALD therapies, as such targets are known to have a significantly higher probability of success in clinical development.

## Conclusion

In conclusion, this study leverages robust genetic methods to de-risk and prioritize four novel causal proteins in ALD. We propose a model where ALD risk is shaped by a balance between pro-inflammatory, matrix-remodeling activities in myeloid cells (mediated by TREML2 and MMP12) and protective, homeostatic functions in hepatocytes (mediated by PLA2R1 and MAX). These findings not only deepen our understanding of ALD pathogenesis but also present genetically validated targets for future therapeutic development.

## Data Availability

The datasets presented in this study can be found in online repositories. The names of the repository/repositories and accession number(s) can be found in the article/**Supplementary Material**.

## Glossary

ALD: Alcohol-Associated Liver Disease
ALT: Alanine Aminotransferase
AST: Aspartate Aminotransferase
ECM: Extracellular Matrix
FDR: False Discovery Rate
GO: Gene Ontology
GWAS: Genome-Wide Association Study
HEIDI: Heterogeneity in Dependent Instruments
IVW: Inverse-Variance Weighted
MAX: MYC Associated Factor X
MMP12: Matrix Metallopeptidase 12
MR: Mendelian Randomization
PLA2R1: Phospholipase A2 Receptor 1
pQTL: protein Quantitative Trait Loci
qPCR: quantitative Polymerase Chain Reaction
scRNA-seq: single-cell RNA sequencing
SMR: Summary-data-based Mendelian Randomization
SNP: Single Nucleotide Polymorphism
sPLA2: secretory Phospholipase A2
TREML2: Triggering Receptor Expressed on Myeloid cells-like 2.
