# Correction: A multi-omics approach combining causal inference and *in vivo* validation identifies key protein drivers of alcohol-associated liver disease

**DOI:** 10.3389/fimmu.2025.1763736

**Published:** 2025-12-15

**Authors:** Qingyi Zhou, Xuan Ma, Qianqian Cui, Lei Zhang, Chao Yao, Zilu Zhang, Xiaoli Wang, Liang Chu

**Affiliations:** 1 Second Affiliated Hospital of Bengbu Medical University, Bengbu, China; 2 Bengbu Medical University, Bengbu, China; 3 Anhui Key Laboratory of Infection and Immunity of Bengbu Medical College, Bengbu, China; 4 School of Clinical Medicine, Shandong Second Medical University, Weifang, Shandong, China; 5 Department of Basic Medical College, Bengbu Medical University, Bengbu, China

**Keywords:** alcohol-associated liver disease, Mendelian randomization, SMR, pQTL, single-cell RNA sequencing, causal inference

The order of affiliations was erroneously listed in the published paper as:

“1Bengbu Medical University, Bengbu, China

2Anhui Key Laboratory of Infection and Immunity of Bengbu Medical College, Bengbu, China

3Second Affiliated Hospital of Bengbu Medical University, Bengbu, China

4School of Clinical Medicine, Shandong Second Medical University, Weifang, Shandong, China

5Department of Basic Medical College, Bengbu Medical University, Bengbu, China”

The correct order of the affiliations is given below:

“1. Second Affiliated Hospital of Bengbu Medical University, Bengbu, China
2. Bengbu Medical University, Bengbu, China
3. Anhui Key Laboratory of Infection and Immunity of Bengbu Medical College, Bengbu, China
4. School of Clinical Medicine, Shandong Second Medical University, Weifang, Shandong, China
5. Department of Basic Medical College, Bengbu Medical University, Bengbu, China”

Correspondingly, the author affiliation superscripts have been updated while keeping the author order unchanged.

The original version of this article has been updated.

