# Supplementary figures and images for: A multi-omics approach combining causal inference and in vivo validation identifies key protein drivers of alcohol-associated liver disease
Source: Front Immunol. 2025 Dec 5;16:1714502. doi: 10.3389/fimmu.2025.1714502 (PMC12767219; doi:10.3389/fimmu.2025.1714502)

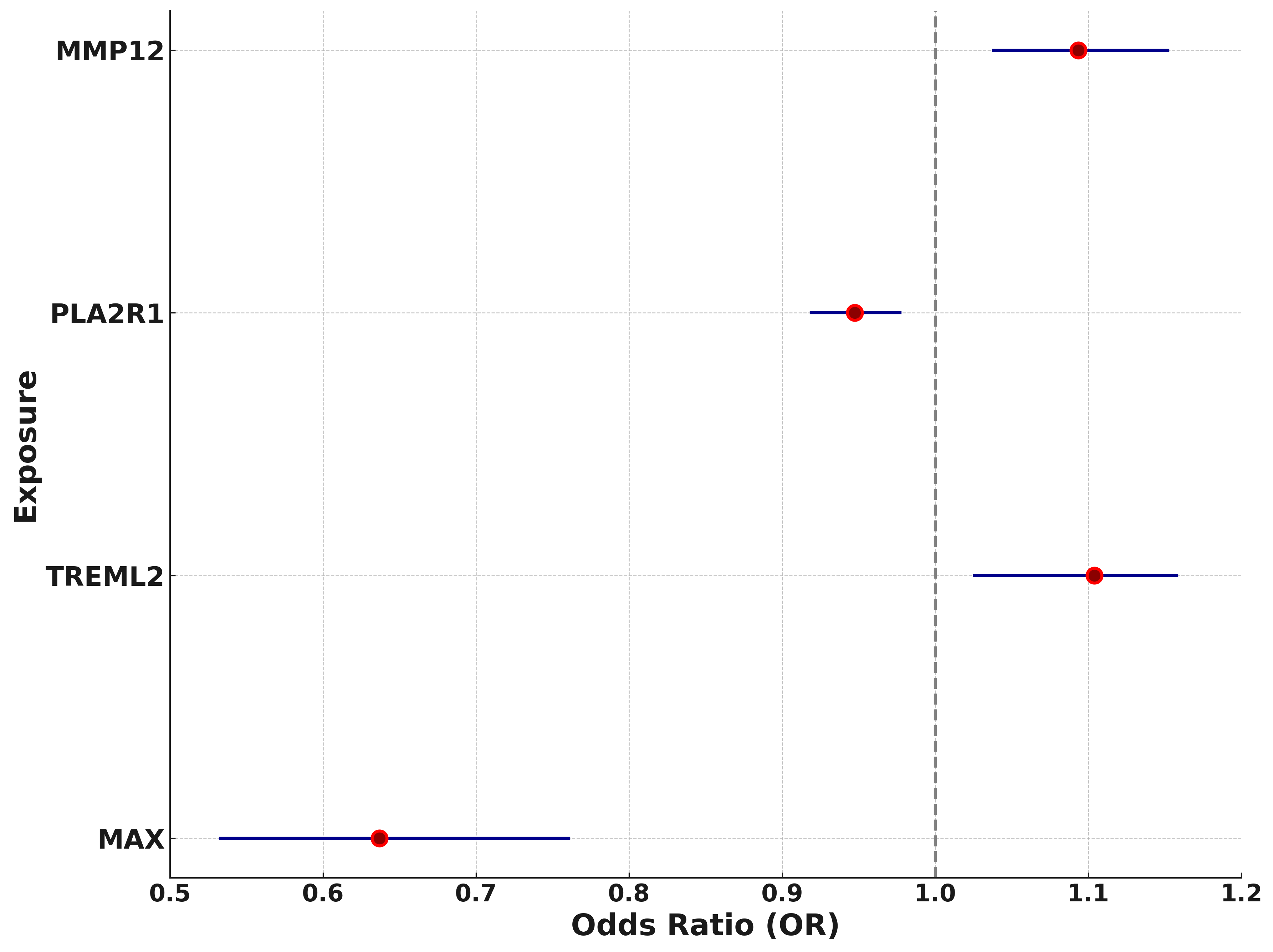

Supplement: Supplementary file 2 [file Image1.jpeg]
