# Supplementary material for: A multi-omics approach combining causal inference and in vivo validation identifies key protein drivers of alcohol-associated liver disease
Source: Front Immunol. 2025 Dec 5;16:1714502. doi: 10.3389/fimmu.2025.1714502 (PMC12767219; doi:10.3389/fimmu.2025.1714502)
Supplement: Supplementary file 4 [file Table2.docx]

# Clear environment

rm(list=ls())

# Check and install required packages

**if**(!**require**("pacman")) install.packages("pacman", update = F, ask = F)

**if** (!**require**("devtools")) install.packages("devtools")

**if** (!**require**("data.table")) install.packages("data.table")

**if** (!**require**("TwoSampleMR")) devtools::install_github("MRCIEU/TwoSampleMR")

# Load necessary libraries

p_load(data.table, dplyr, tidyr, ggplot2, ieugwasr, MRInstruments, plyr)

# Set Bioconductor mirror to USTC

options(BioC_mirror="https://mirrors.ustc.edu.cn/bioc/")

# Create necessary directories

dirs <- c("ORdata", "Pleiotropydata", "Result", "heterogeneity", "PRESSO", "harmonise")

**for** (dir **in** dirs) {

**if** (!dir.exists(dir)) dir.create(dir)

}

# Get the list of CSV files in the 'pQTL' folder

FileNames <- list.files(path = "pQTL/", pattern = "*.csv")

FileNames

# Initialize variables for storing exposure data

exp_dat <- list()

ex_pore <- c()

# Process exposure data files

**for**(i **in** seq_along(FileNames)) {

**tryCatch**({

# Read exposure data

IV <- fread(paste0("pQTL/", FileNames[i]))

IV$PHENO <- FileNames[i] # Add phenotype column

# Format data to the required TwoSampleMR format

IV1 <- format_data(IV,

type = "exposure",

phenotype_col = "PHENO",

id_col = "gene",

snp_col = "SNP",

beta_col = "BETA",

se_col = "SE",

eaf_col = "EAF",

effect_allele_col = "effect_allele",

other_allele_col = "other_allele",

pval_col = "P",

samplesize_col = "samplesize",

chr_col = "CHR",

pos_col = "BP")

# Store formatted data

exp_dat[[i]] <- IV1

ex_pore <- c(ex_pore, FileNames[i])

}, error = **function**(e) {

cat("Error occurred for file:", FileNames[i], "\n")

cat("Error message:", conditionMessage(e), "\n")

cat("Skipping this file...\n")

})

}

# Save exposure data

save.image("exposure.Rdata")

load("exposure.Rdata")

# Outcome data processing

outcomefile <- "ald.csv" # Modify this for your own data

deseasename <- "ALD"

GWAS_1 <- vroom::vroom(outcomefile)

# Bind all exposure data and filter GWAS data for common SNPs

allSNP <- do.call(rbind, exp_dat)

GWAS_2 <- subset(GWAS_1, GWAS_1$SNP %**in**% allSNP$SNP)

rm(GWAS_1) # Free memory

GWAS_2$PHENO <- deseasename

# Format outcome data

out_data <- format_data(

GWAS_2,

type = "outcome",

phenotype_col = "PHENO",

snp_col = "SNP",

beta_col = "BETA",

se_col = "SE",

eaf_col = "EAF",

pval_col = "P",

effect_allele_col = "effect_allele",

other_allele_col = "other_allele",

chr_col = "CHR",

pos_col = "BP",

samplesize_col = "samplesize"

)

# Store outcome data in a list

out_dat <- list(out_data)

out_come <- deseasename

# Save outcome data

save.image("outcome.Rdata")

load("outcome.Rdata")

# Initialize list for results

results <- list()

# Perform MR analysis for each combination of exposure and outcome

**for** (i **in** seq_along(ex_pore)) {

**for** (j **in** seq_along(out_come)) {

**tryCatch**({

# Harmonize data

dat <- harmonise_data(

exposure_dat = exp_dat[[i]],

outcome_dat = out_dat[[j]],

action = 1

)

dat <- subset(dat, mr_keep == TRUE) # Keep valid data

# Calculate R2 and f statistics

dat$R2 <- (2 * (dat$beta.exposure^2) * dat$eaf.exposure * (1 - dat$eaf.exposure)) /

(2 * (dat$beta.exposure^2) * dat$eaf.exposure * (1 - dat$eaf.exposure) +

2 * dat$samplesize.exposure * dat$eaf.exposure * (1 - dat$eaf.exposure) * dat$se.exposure^2)

dat$f <- dat$R2 * (dat$samplesize.exposure - 2) / (1 - dat$R2)

dat$meanf <- mean(dat$f)

dat <- dat[dat$f > 10,] # Filter for valid data

# MR analysis

res <- mr(dat)

result_or <- generate_odds_ratios(res)

# If p-value is less than the threshold, save results

**if**(result_or$pval[3] < pfilter) {

filename <- basename(sub("\\.txt$", "", ex_pore[i]))

filename2 <- sub("\\.csv$", "", filename)

# Create output directories

dir.create(paste0("./Result/", filename2))

# Save results

write.table(dat, file = paste0("./Result/", filename2, "/harmonise.csv"), row.names = F, sep = ",", quote = F)

write.table(dat, file = paste0("./harmonise/", filename2, "_harmonise.csv"), sep = ",", quote = F, row.names = F)

write.table(result_or, file = paste0("./Result/", filename2, "/OR.csv"), row.names = F, sep = ",", quote = F)

write.table(result_or, file = paste0("./ORdata/", filename2, "_OR.csv"), row.names = FALSE, sep = ",", quote = F)

# Generate and save plots

p1 <- mr_scatter_plot(res, dat)

ggsave(p1[[1]], file = paste0("./Result/", filename2, "/scatter.pdf"), width = 8, height = 8)

# Perform pleiotropy test

pleiotropy <- mr_pleiotropy_test(dat)

write.table(pleiotropy, file = paste0("./Result/", filename2, "/pleiotropy.csv"), sep = ",", quote = F, row.names = F)

write.table(pleiotropy, file = paste0("./Pleiotropydata/", filename2, "_pleiotropy.csv"), sep = ",", quote = F, row.names = F)

# Perform heterogeneity test

heterogeneity <- mr_heterogeneity(dat)

write.table(heterogeneity, file = paste0("./Result/", filename2, "/heterogeneity.csv"), sep = ",", quote = F, row.names = F)

write.table(heterogeneity, file = paste0("./heterogeneity11/", filename2, "_heterogeneity.csv"), sep = ",", quote = F, row.names = F)

# Run MR-PRESSO

presso <- run_mr_presso(dat, NbDistribution = 1000)

capture.output(presso, file = paste0("./Result/", filename2, "/presso.csv"))

write.table(presso[[1]]$`Main MR results`, file = paste0("./PRESSO/", filename2, "_mrPRESSO_Main_MR_results.csv"), sep = ",", quote = F, row.names = F)

write.table(presso[[1]]$`MR-PRESSO results`$`Global Test`$Pvalue, file = paste0("./PRESSO/", filename2, "_mrPRESSO_Global_Test_Pvalue.csv"), sep = ",", quote = F, row.names = F)

# Single-SNP analysis

singlesnp_res <- mr_singlesnp(dat)

singlesnpOR <- generate_odds_ratios(singlesnp_res)

write.table(singlesnpOR, file = paste0("./Result/", filename2, "/singlesnpOR.csv"), row.names = F, sep = ",", quote = F)

# Forest plot

p2 <- mr_forest_plot(singlesnp_res)

ggsave(p2[[1]], file = paste0("./Result/", filename2, "/forest.pdf"), width = 8, height = 8)

# Leave-one-out analysis

sen_res <- mr_leaveoneout(dat)

p3 <- mr_leaveoneout_plot(sen_res)

ggsave(p3[[1]], file = paste0("./Result/", filename2, "/sensitivity-analysis.pdf"), width = 8, height = 8)

# Funnel plot test

p4 <- mr_funnel_plot(singlesnp_res)

ggsave(p4[[1]], file = paste0("./Result/", filename2, "/funnelplot.pdf"), width = 8, height = 8)

# Store exposure and outcome names in results

res$exposure <- ex_pore[i]

res$outcome <- out_come[j]

# Store result

results[[length(out_come)*(i-1)+j]] <- generate_odds_ratios(res)

}

}, error = **function**(e) {

cat("Error occurred for exposure:", ex_pore[i], ", outcome:", out_come[j], "\n")

cat("Error message:", conditionMessage(e), "\n")

cat("Skipping this combination...\n")

})

}

}

# Combine all results and save to CSV

results_allIV <- do.call(rbind, results)

fwrite(results_allIV, "result.csv")

**library**(MendelR)

config_data_mode(3)

a = data.table::fread("qtl_map.csv")

smr_run_make_qtl(ids=a$id, Genes=a$gene, out_beqtl_name="test5",

pval_threshold=5e-8, cis_wind=1000,

build_version="hg38",

id_outcomes=NULL, recreate=F)

smr_run_whole("gwas.ma", beqtl_file = "qtl_files/test5")
